# Psychological states could affect postsurgical pain after hemorrhoidectomy: A prospective cohort study

**DOI:** 10.3389/fsurg.2022.1024237

**Published:** 2023-01-06

**Authors:** Geng Wang, Yuanjue Wu, Yang Cao, Rui Zhou, Kaixiong Tao, Linfang Wang

**Affiliations:** ^1^ Department of Gastrointestinal Surgery, Union Hospital, Tongji Medical College, Huazhong University of Science and Technology, Wuhan, China; ^2^ Department of Clinical Nutrition, Union Hospital, Tongji Medical College, Huazhong University of Science and Technology, Wuhan, China; ^3^ Cancer Center, Union Hospital, Tongji Medical College, Huazhong University of Science and Technology, Wuhan, China

**Keywords:** hemorrhoids, postsurgical pain, psychological states, hemorrhoidectomy, depression

## Abstract

**Background:**

Open hemorrhoidectomy is one of the standard procedures for grade IV hemorrhoids. Postsurgical pain is a common problem for patients. We aim to prospectively evaluate potential factors affecting postoperative pain among hemorrhoidectomy patients.

**Methods:**

An observational study was conducted on 360 patients who had undergone Milligan-Morgan open hemorrhoidectomy. Details of the surgery and baseline information were recorded. Preoperative anxiety and depression were analyzed via the self-rating anxiety scale 20 (SAS-20) and self-rating depression scales 20 (SDS-20), respectively. Postoperative pain score was performed daily after surgery until the patient was discharged. The numerical pain score was evaluated by the visual analogue scale (VAS). The association between preoperative psychological states (anxiety or depression) and postoperative pain was analyzed using a generalized additive mixed model.

**Results:**

A total of 340 patients eventually provided complete data and were included in our study. The average age was 43.3 ± 14.4 years, and 62.1% of patients were women. In total, 14.9% of patients had presurgical anxiety and 47.1% had presurgical depression. Postsurgical pain reached a peak point 1–2 days after surgery and went down to a very low level around 4–5 days after surgery. More excision of hemorrhoids could lead to more pain experience after surgery. Presurgical depression was associated with postsurgical pain. Patients who had presurgical depression had higher pain scores after surgery (2.3 ± 1.9 vs. 3.3 ± 1.9, *p* = 0.025).

**Conclusion:**

Preoperative depression and the amount of excisional hemorrhoids are positively related to postsurgical pain.

## Introduction

Hemorrhoid disease is one of the most frequent complaints affecting a large population. In China, the prevalence rates were around 18% in the elderly population (1). Some researchers reported that 13.1% of all patients in the surgical outpatient department had hemorrhoids (2). According to the guideline statements from different countries such as Italy (3), Japan (4), and many other countries (5–7), for patients with grade IV hemorrhoids, Milligan-Morgan open hemorrhoidectomy is still one of the standard procedures (8). The frequently encountered one after open hemorrhoidectomy is postsurgical pain (9, 10). In clinical practice, the size of the surgery area and the psychological states may all affect the degree of postsurgical pain (11). However, we still lack related data.

Previous studies have shown that emotional distress, such as presurgical anxiety and depression, is positively correlated with postsurgical outcomes (12, 13). In surgeries such as knee arthroscopy, bariatric surgery, and hip fracture repair, significant psychological influences on hospital stays and costs were reported (14). There are studies suggesting that the presence of preoperative anxiety is usually associated with poorer quality of life and cognitive performance (15). Also, patients with depression had worse perceptions of their shared decision-making process with their surgeon (10). However, in patients with hemorrhoids, the relationship between preoperative anxiety/depression and the development of postoperative pain has not been studied sufficiently.

We initiated this observational study to analyze which factors could affect subjective pain after surgery. Both the subjective and objective factors were taken into consideration. We aimed to figure out factors related to the degree of postsurgical pain. With the findings, we could predict the degree of pain after hemorrhoidectomy for everyone. Personalized pain management could then be adopted.

## Patients and methods

**Figure 1:**
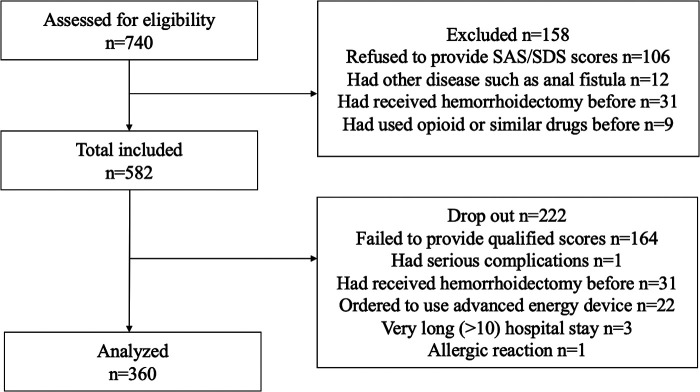
.

This is a prospective study. From January 2019 to December 2021, 740 patients with a symptomatic fourth degree of hemorrhoids underwent Milligan-Morgan open hemorrhoidectomy. Patients who did not reach fourth-degree hemorrhoids were excluded. All patients had signed written informed consent. The local ethics committee and departmental internal review board approved this study. Patients who could not cooperate with data collection or had other postsurgical complications were excluded. Patients who had used opioids before admission to the hospital were excluded. Incidences of complications during the hospital stay period were recorded. Patients with serious complications shortly after surgery were excluded. Finally, 360 patients finished the study and provided eligible scales. Details are shown in Figure 1.

This is an observational study. Symptom onset, pain score, analgesic consumption, duration of hospital stay, and complications were recorded. A detailed preoperative consultation with a comprehensive information booklet for hemorrhoidectomy was provided to all the patients. The same surgeon performed all surgery. We did not use local anesthesia before surgery. The number of removed packs is listed in Table 2. High-frequency elytrotomy was used to stop bleeding during the surgery. Routinely, a single intradermal injection of methylene blue and ropivacaine was given to all patients right after surgery. Two millilters of 1% methylene blue and 8 ml of 10 mg/ml ropivacaine were mixed and injected into the surgical area (16, 17). We did not place a plug in the anal canal after surgery. The details of the surgery were immediately recorded after the operation. A standardized general anesthetic technique using propofol, fentanyl, and inhalation anesthesia was used. Laxatives were used the night before surgery, and oral enteral nutrition was used from the first day after surgery until the patients were discharged (4–5 days after surgery). An oral analgesic (tramadol hydrochloride sustained release tablets) was given to the patients after surgery. Patients may take a pill when they feel hard to bear.

## Assessment of psychological status

To assess whether psychological factors play a role, all patients were asked to finish the anxiety and depression questionnaire before surgery. Self-rating anxiety scale 20 (SAS 20) and self-rating depression scale 20 (SDS 20) were used to quantify the psychological status. High score values indicate high levels of anxiety or depression. By referring to the Chinese national norm, an SAS score <50 was considered normal for anxiety (18). An SDS score <53 was considered normal for depression (18).

## Evaluation of postsurgical pain

Postsurgical pain was assessed every day after surgery. The visual analogue pain score system was used (19). Every day, a 10-cm linear analogue scale was used to assess. We evaluated the pain score three times a day and recorded the worst pain experienced on the day. The total number of analgesic tablets taken during the day was also recorded.

## Statistical analysis

Baseline characteristics are presented as means ± SDs for normally distributed variables, medians (interquartile ranges) for non-normally distributed variables, and frequencies and percentages for categorical variables. Analysis of variance, the Kruskal–Wallis rank test, and the chi-square test were used to compare characteristics, clinical data, and postoperative pain scores where appropriate. In this study, longitudinal data were postoperative pain scores over time. The longitudinal changes in postoperative pain scores were analyzed using the generalized additive mixed model (GAMM), which is ideal for longitudinal data analysis because it is easy to accommodate unbalanced and unevenly spaced observations (20, 21). In these models, the dependent variable (i.e., postsurgical pain) was assessed during all follow-up visits, whereas the independent variables were only measured on the baseline visit. All models also included intercept and time as random terms. Random effects allowed each participant's starting value to vary from the population average (intercept) and the longitudinal trajectory to vary from the population average longitudinal trajectory (slope). All analyses were performed with R software version 3.4.3 (http://www.R-project.org; The R Foundation) and EmpowerStats version 2.20 (http://www.empowerstats.com; X&Y Solutions, Inc., Boston, MA). A *P*-value <0.05 was considered statistically significant (two-sided tests).

## Results

### Clinical characteristics

Finally, 340 patients provided qualified VAS scores and SAS/SDS questionnaires without any postsurgical complications. Table 1 shows the clinical baselines of the patients. The average age was 43.3 ± 14.4 years. There are 211 women (62.1%) and 129 men (37.9%) in the study. Fifty-one patients (14.9%) had different levels of anxiety, and 160 patients (47.1%) had different levels of depression. Ninety-one patients (26.7%) received excision of one external hemorrhoid, 119 patients (35.0%) received two, 125 patients (36.8%) received three, and 5 (1.4%) received four. Twenty-two patients (6.1%) had continence problems, 1 patient (0.2%) had slight postoperative bleeding, and 15 patients (4.2%) had urine retention. Details are shown in Table 1 and Figure 1.

**Table 1:** Demographics and clinical data at baseline.

Variables	Results
	Mean ± SD
Age, years	43.3 ± 14.4
BMI, kg/m^2^	23.0 ± 3.0
HB, g/L	121.9 ± 24.7
ALB, g/L	43.4 ± 4.2
Sex, *n* (%)	
Female	211 (62.1%)
Male	129 (37.9%)
Education	
Illiteracy	31 (9.2%)
Elementary school	35 (10.3%)
Middle/high school	88 (26.4%)
University	184 (54.0%)
Unclear	31 (9.2%)
Smoking, yes, *n* (%)	12 (3.4%)
Drinking, yes, *n* (%)	27 (2.3%)
Diabetes, yes, *n* (%)	0 (0%)
Hypertension, yes, *n* (%)	27 (8.0%)
CVD, yes, *n* (%)	4 (1.1%)
Number of excisional hemorrhoids, *n* (%)	
1	91 (26.7%)
2	119 (35.0%)
3	125 (36.8%)
≥4	5 (1.4%)
Hematochezia, yes, *n* (%)	266 (78.2%)
Serious prolapse, yes, *n* (%)	235 (69.0%)
Anxiety, *n* (%)	
No (SAS score <50)	289 (85.1%)
Mild (SAS score 50–59)	35 (10.3%)
Moderate (SAS score 60–69)	16 (4.6%)
Severe (SAS score ≥70)	0 (0%)
Depression, *n* (%)	
No (SDS score <53)	180 (52.9%)
Mild (SDS score 53–62)	152 (44.8%)
Moderate (SDS score 63–71)	8 (2.3%)
Severe (SDS score ≥72)	0 (0%)

BMI, body mass index; HB, hemoglobin; ALB, albumin; CVD, cardiovascular disease; SAS, self-rating anxiety scale; SDS, self-rating depression scale.

### Postsurgical pain scores

The VAS system was used to quantify the pain degree. Pain score was the highest on the first day after surgery and slowly decreased afterward. On days 4–5, the degree of pain returned to a very low level. Patients were usually discharged on the fifth day after surgery. Details are shown in Table 2.

**Table 2:** Demographic, clinical data, and postoperative pain score according to anxiety or depression.

Variables	Anxiety			Depression		
	No	Yes	*p*-Value	No	yes	*p*-Value
	289	51		180	160	
Age, years	44.2 ± 14.6	38.2 ± 12.3	0.165	45.9 ± 16.2	40.4 ± 11.5	0.074
BMI, kg/m^2^	23.2 ± 3.1	22.3 ± 2.3	0.327	23.2 ± 3.3	22.9 ± 2.6	0.684
HB, g/L	120.7 ± 26.1	128.9 ± 11.6	0.289	121.7 ± 26.0	122.2 ± 23.3	0.925
ALB, g/L	43.3 ± 4.4	43.8 ± 3.4	0.688	43.2 ± 4.1	43.6 ± 4.5	0.704
Sex, *n* (%)			0.564			0.492
Female	176 (60.8%)	35 (69.2%)		106 (58.7%)	105 (65.9%)	
Male	113 (39.2%)	16 (30.8%)		74 (41.3%)	55 (34.1%)	
Education			0.428			0.046
Illiteracy	27 (9.5%)	4 (7.7%)		23 (13.0%)	8 (4.9%)	
Elementary school	31 (10.8%)	4 (7.7%)		20 (10.9%)	16 (9.8%)	
Middle/high school	55 (18.9%)	4 (7.7%)		16 (8.7%)	43 (26.8%)	
University	145 (50.0%)	10 (76.9%)		113 (63.0%)	70 (43.9%)	
Unclear	31 (10.8%)	0 (0.0%)		8 (4.3%)	23 (14.6%)	
Smoking, yes, *n* (%)	12 (4.1%)	0 (0.0%)	0.46	8 (4.3%)	4 (2.4%)	0.626
Drinking, yes, *n* (%)	8 (2.7%)	0 (0.0%)	0.549	4 (2.2%)	4 (2.4%)	0.934
Hypertension, yes, *n* (%)	23 (8.1%)	4 (7.7%)	0.959	22 (15.2%)	5 (0.0%)	0.069
CVD, yes, *n* (%)	4 (1.4%)	0 (0.0%)	0.673	4 (2.2%)	0 (0.0%)	0.342
Number of excisional hemorrhoids, *n* (%)		0.476			0.84	
1	80 (27.7%)	11 (21.6%)		39 (21.7%)	52 (32.5%)	
2	95 (32.9.1%)	24 (47.1%)		63 (34.8%)	56 (35.0%)	
≥3	114 (39.4%)	16 (31.4%)		78 (43.5%)	52 (32.5%)	
Hematochezia, yes, *n* (%)	219 (75.7%)	47 (92.3%)	0.181	141 (78.3%)	125 (78.0%)	0.981
Serious prolapse, yes, *n* (%)	199 (68.9%)	35 (69.2%)	0.982	125 (69.6%)	109 (68.3%)	0.898
Postoperative pain score						
POD1	4.3 ± 2.3	4.1 ± 2.5	0.71	4.2 ± 2.2	4.4 ± 2.4	0.8
POD2	3.1 ± 1.9	3.7 ± 1.5	0.286	2.9 ± 2.0	3.5 ± 1.6	0.169
POD3	2.6 ± 2.0	3.6 ± 1.8	0.09	2.3 ± 1.9	3.3 ± 1.9	0.025
POD4	2.4 ± 1.7	3.0 ± 2.1	0.259	2.1 ± 1.8	2.9 ± 1.7	0.05
POD5	2.4 ± 1.8	2.6 ± 1.4	0.713	2.4 ± 2.0	2.4 ± 1.4	0.911

BMI, body mass index; HB, hemoglobin; ALB, albumin; POD, post operation days; CVD, cardiovascular disease.

### Consumption of oral analgesic

The total amount of oral analgesic consumed by the patients after surgery was recorded. Patients with anxiety took a higher amount of pain killer 2–3 days after surgery (day 2: 1.5 ± 0.7 vs. 3.4 ± 0.8, *p* = 0.006; day 3: 1.5 ± 4.4 vs. 2.5 ± 1.2, *p* = 0.03). Details are shown in Table 3.

**Table 3:** The amount of oral analgesic consumed by the patients during hospital stay after surgery.

Variables	Anxiety			Depression		
	No	Yes	*p*-Value	No	yes	*p*-Value
No. of patients	289	51		180	160	
No. of pills consumed						
POD1	2.8 ± 1.1	2.9 ± 0.9	0.412	2.9 ± 1.0	2.8 ± 1.1	0.922
POD2	1.5 ± 0.7	3.4 ± 0.8	0.006	1.7 ± 0.6	1.9 ± 0.8	0.312
POD3	1.5 ± 1.4	2.5 ± 1.2	0.03	1.5 ± 0.5	1.8 ± 0.7	0.074
POD4	1.6 ± 0.5	1.7 ± 0.6	0.728	1.4 ± 0.3	1.5 ± 0.5	0.522
POD5	0.9 ± 0.4	1.1 ± 0.5	0.922	0.8 ± 0.6	1.0 ± 0.4	0.621

POD, postoperation day.

Data are shown as mean ± SD.

### Preoperative psychological characteristics and postsurgical pain

The preoperative feeling of anxiety and depression was evaluated by SAS 20 and SDS 20. Before surgery, in 340 patients, 51 patients (14.9%) had abnormal levels of anxiety. A total of 160 patients (47.05%) were assessed to have depression. Details are shown in Table 2. Gender, education, and the number of external hemorrhoids did not differ between the normal and abnormal groups, except that patients with a higher education level seem to have a higher burden of depression (*p* = 0.046). There were no significant differences in terms of pain scores between patients with and without anxiety. On the third day after surgery, patients with depression had significantly higher pain scores (2.3 ± 1.9 vs. 3.3 ± 1.9, *p* = 0.025).

### Other characteristics correlated to postsurgical pain

We recorded the number of excisional hemorrhoids. We also studied the educational background, alcohol/tobacco consumption, and clinical symptoms such as prolapsus and hematochezia. Patients who underwent more hemorrhoid excision had higher pain scores after surgery. No differences were found in terms of other factors. Details are shown in Supplementary Table S1.

### Association between preoperative psychological state and postoperative pain

The GAMM model showed that the postoperative pain score decreased significantly with time in the crude model (*β*: −0.52; 95% CI: −0.63 to −0.41; *p* < 0.001) and fully adjusted model (*β*: −0.53; 95% CI: −0.65 to −0.42; *p* < 0.001). In the crude model (model 1), compared with patients without depression, those with depression have higher postoperative pain. However, it was not statistically significant (*β*: 0.50; 95% CI: −0.12 to 1.11; *p* = 0.118). In model 2 adjusted for age, sex, education, and patients with depression were independently and significantly associated with postoperative pain (*β*: 0.77; 95% CI: −0.13 to 1.41; *p* = 0.021). In the full model (model 3), further adjusted fo the number of excisional haemorrhoids, the association between depression and postoperative pain was close to the margin of statistical significance (*β*: 0.61; 95% CI: 0.01 to 1.21; *p* = 0.05). However, we did not observe significant anxiety and postoperative pain in all models. Details are shown in Table 4.

**Table 4:** Preoperative anxiety and depression and postoperative pain score in a linear mixed-effects regression model.

	Model 1		Model 2		Model 3	
	*β* (95%CI)	*p* value	*β* (95%CI)	*P* value	*β* (95%CI)	*p* value
Preoperative anxiety						
Anxiety	0.53 (−0.33, 1.39)	0.228	0.57 (−0.29, 1.43)	0.2	0.40 (−0.40, 1.21)	0.328
Time	−0.52 (−0.63, −0.41)	<0.001	−0.52 (−0.63, −0.41)	<0.001	−0.53 (−0.65, −0.42)	<0.001
Preoperative depression						
Depression	0.50 (−0.12, 1.11)	0.118	0.77 (0.13, 1.41)	0.021	0.61 (0.01, 1.21)	0.05
Time	−0.52 (−0.63, −0.41)	<0.001	−0.52 (−0.63, −0.41)	<0.001	−0.53 (−0.65, −0.42)	<0.001

Model 1: Crude model.

Model 2: adjusted for age, sex. and education.

Model 3: further adjusted for the number of excisional hemorrhoids.

## Discussion

In this study, we aimed to study the effect of psychological state on postsurgical pain after open hemorrhoidectomy. Since the factors affecting pain are too complicated, we set several restrictions. Only patients with fourth-degree hemorrhoids were recruited for our research. Also, patients who had taken opioids or other psychotropic drugs before admission to the hospital were excluded. Thus, the patients had similar conditions before surgery. Patients with serious postsurgical complications (serious infection, anal fissure, anorectal stenosis, serious bleeding) were excluded. We found that the pain score was highest on the first day after surgery and slowly decreased afterward. The GAMM model showed that the postoperative pain score decreased significantly with time in the crude model. There are no significant differences in terms of pain scores between patients with and without anxiety. However, patients with anxiety took higher amounts of pain killer 2–3 days after surgery. On the other hand, patients with depression had significantly higher pain scores on the third day after surgery than others. In the fully adjusted model adjusted for age, sex, education, and the number of excisional hemorrhoids, we still found that patients with depression were independently and significantly associated with postoperative pain. Together, our data suggested that depression could promote postsurgical pain after Milligan-Morgan open hemorrhoidectomy.

Before we started this research, we analyzed the sample size using statistical Software G*Power 3.1.7 for Windows (https://www.psychologie.hhu.de/arbeitsgruppen/allgemeine-psychologie-und-arbeitspsychologie/gpower.html). Considered an effect size (*f*) of 0.30, error probability alpha of 0.05, power (1 – error probability beta) of 0.90, the number of groups of 2, the number of measurements of 5, a correlation among repeated measures of 0.5 for *F* tests (ANOVA: repeated measures), the calculated total sample size was 74. We finally had 340 patients. In addition, a post hoc power analysis was conducted by using the same statistical software. Considering an effect size (*f*) of 0.30, error probability alpha of 0.05, the total sample size of 340, the number of groups of 2, the number of measurements of 5, a correlation among repeated measures of 0.5 for *F* tests (ANOVA: repeated measures), the calculated power (1 – error probability beta) was 0.95. Thus, based on the results of priori power analyses and posthoc power analyses, we believe that this study has an adequate sample size.

In this study, we chose to perform Milligan-Morgan open hemorrhoidectomy. Milligan-Morgan surgery is a classic surgery and is widely performed (22). This surgery has a very low medical cost and recurrence rate. Although new techniques such as stapled hemorrhoidopexy and DGHAL did reduce postsurgical pain, they required higher medical fees and had higher recurrence rates for grade IV hemorrhoids (23). Restriction of the medical fee also restricts the use of the type of energy. The LigaSure system and Ultrasonic Scalpel were recommended in many studies (24, 25). We had used the device in some cases. The clinical results were excellent. These equipment could reduce operation time, blood loss, and postoperative pain (26). However, due to the limitation of the medical insurance policy, to reduce the medical cost to meet the demand of Diagnosis-Related Groups (DRGs), we only used the normal high-frequency elytrotomy to stop bleeding during hemorrhoidectomy in all the cases in our study.

In our study, we used VAS to investigate postsurgical pain. It is very important in terms of sensitivity and the ability to detect changes in pain over time. VAS is more valid for detecting changes in pain intensity (27). That is why we chose VAS in our study. Also, in many similar studies, VAS was also used to examine postsurgical pain after hemorrhoidectomies (28, 29). Indeed, there are limitations to the self-reporting scales. There are many other scales for rating anxiety and depression. Both SAS/SDS 20 and HADS were widely used, and many clinical studies adopted either of the scales. We tried both scales. HADS contained fewer questions and occupied less time. However, we found that there are two problems. First, sometimes, the patients were not careful enough and did not provide accurate answers. Second, many patients were elderly people. They could not understand the questions in the HADS. SAS/SDS 20, however, had a design of reverse scoring. We could check the scale and found it obvious. The patients were then asked to finish the scale again. In this way, the scores were more reliable. Thus, we chose SAS/SDS 20 for our study.

In our study, the median pain score was the highest on day 1. Some studies mentioned that many patients had defecatory pain after surgery. This happened around days 3–4 after surgery (30). In our medical center, we used laxatives before surgery and oral enteral nutrition after surgery instead of a normal diet. This largely retarded the defecation time after surgery, and we did not observe significant pain complaints during this period.

We found that patients with fewer excisional hemorrhoids had less pain 1 and 2 days after surgery (*p* = 0.021, *p* = 0.002). This finding was not surprising because more excisional hemorrhoids would lead to more surgical trauma in the local site. Also, this would cause postoperative spasm of the anal sphincter and leave the inflammation reaction around the anal. Thus, unlike malignant diseases, the surgical concept for hemorrhoids should be as less as possible. Also, we validated the affection of pre-surgical symptoms, such as hematochezia and prolapsus. Moreover, the results were negative.

In addition to the surgery itself, the perception and the psychological status of the patient are also very important. Previous studies suggested that anxiety and depression prior to surgery were correlated with postsurgical pain (12). These patients had higher analgesic requirements. Moreover, pain may also aggravate the status of depression/anxiety. These changes may trigger each other and finally lead to a vicious circle. We found that patients with anorectal disease usually had abnormal psychological states. Our data suggested that 14.9% of all patients had different levels of anxiety and 47.1% had different levels of depression. It seemed that patients with grade IV hemorrhoids properly had depression.

To study whether anxiety and depression may affect postsurgical pain, the generalized additive mixed model was used for the study. In a systematic review, 8 studies reported a significantly effect and 10 studies did not demonstrate a significant difference between patients with and without depression (15). In our study, we did not observe significant differences in terms of anxiety. However, on the other hand, patients with depression had prolonged pain duration. After excluding the effects of age, gender, education, and the number of excisional hemorrhoids, patients with depression still had higher pain scores. Our finding suggested that presurgical depression did contribute to more pain complaints after surgery. Depression is commonly associated with cognitive impairment, and this could lower the threshold for acute postoperative pain (31). Also, in depression disorders, the immune system of the patient is suppressed and there might be higher chances of infection. After hemorrhoidectomy, this might promote pain.

We also recorded the amount of oral analgesic each patient taken during their hospital stay. Interestingly, it seemed that although patients with anxiety did not have higher pain scores, they consumed higher amounts of the drugs than others (supplementary Table S2). Since the effects of the drug could last for 4–6 h, the worst pain experience of the day could still be recorded and not affected. This finding suggested that patients with anxiety seemed to take more drugs than other patients under similar pain burdens.

In our study, the length of hospital stay (LOS) was usually 4–5 days after surgery. The LOS varied very much due to the different situations in different countries. Some surgeons even showed that the LOS could be nearly a month (25). Other reports from China indicated that LOS is 7–13 days (32). Others suggested that it is usually 1–2 days (28). Some groups even performed the surgery in the outpatient setting (33). Despite the fact that there are big differences in terms of LOS, other studies and our groups agreed that it would take almost 7–30 days for the patients to return to normal activity (34, 35). Those who left hospital very early after surgery still require medical support after they went home. It seems that in a place with less community medical support, patients chose to stay in the hospital for longer.

There are several limitations to our study. First, we only record the pain score of hospitalized patients. Thus, we lack the related data when patients were discharged. Second, this is an observational study, and there is no additional intervention treatment. More study is required to figure out advanced therapies. Also, patients with different expectations of the operation may have different readouts in terms of pain degree. As a result, the pain score from the patients may not be very accurate. To weaken the affection, we only recorded the data on patients with symptomatic fourth-degree hemorrhoids. Still, this is not objective enough and advanced methods are required for further study. In addition, there are now advanced devices to help reduce postsurgical pain and there are many reports. In our study, due to the restriction of medical insurance and Diagnosis-Related Groups (DRGs), we only use the normal high-frequency elytrotomy. The results may change when an advanced device is adopted.

In conclusion, our study suggested that the amount of excisional hemorrhoids correlates with the pain of postsurgical pain. In addition, psychological states could also affect the complaints of postsurgical pain. Presurgical depression could increase the degree of postsurgical pain.

## Data Availability

The original contributions presented in the study are included in the article/**Supplementary Material**; further inquiries can be directed to the corresponding author.
