# Supplementary material for: Psychological states could affect postsurgical pain after hemorrhoidectomy: A prospective cohort study
Source: Front Surg. 2023 Jan 6;9:1024237. doi: 10.3389/fsurg.2022.1024237 (PMC9852767; doi:10.3389/fsurg.2022.1024237)
Supplement: Supplementary file 1 [file Table1.docx]

| pre-surgical hematochezia | no | yes | P-value |  |
| --- | --- | --- | --- | --- |
| N | 74 | 266 |  |  |
| POD1 | 3.7 ± 2.2 | 4.5 ± 2.3 | 0.233 |  |
| POD2 | 3.1 ± 1.8 | 3.2 ± 1.8 | 0.837 |  |
| POD3 | 2.8 ± 2.0 | 2.8 ± 2.0 | 0.897 |  |
| POD4 | 2.1 ± 1.7 | 2.6 ± 1.8 | 0.257 |  |
| POD5 | 2.0 ± 1.9 | 2.5 ± 1.6 | 0.314 |  |
|  |  |  |  |  |
|  |  |  |  |  |
| pre-surgical prolapse | no | yes | P-value |  |
| N | 106 | 234 |  |  |
| POD1 | 4.4 ± 2.3 | 4.3 ± 2.3 | 0.848 |  |
| POD2 | 3.4 ± 1.7 | 3.1 ± 1.9 | 0.584 |  |
| POD3 | 3.2 ± 2.0 | 2.6 ± 2.0 | 0.24 |  |
| POD4 | 2.9 ± 1.8 | 2.4 ± 1.8 | 0.269 |  |
| POD5 | 2.4 ± 1.5 | 2.4 ± 1.8 | 0.957 | · |
|  |  |  |  |  |
|  |  |  |  |  |
| Number of excisional hemorrhoids | 1 | 2 | ≥3 | P-value |
| N | 91 | 119 | 130 |  |
| POD1 | 3.1 ± 1.3 | 4.2 ± 2.1 | 4.9 ± 2.6 | *0.021 |
| POD2 | 1.7 ± 1.2 | 3.3 ± 1.7 | 3.7 ± 1.9 | *0.002 |
| POD3 | 1.5 ± 1.5 | 3.0 ± 1.8 | 3.0 ± 2.1 | *0.031 |
| POD4 | 1.4 ± 1.5 | 2.7 ± 1.7 | 2.7 ± 1.9 | 0.168 |
| POD5 | 0.4 ± 0.9 | 3.0 ± 1.6 | 2.3 ± 1.6 | *0.004 |
|  |  |  |  |  |
| (N) Mean+SD / N(%)  POD: post operation days |  |  |  |  |
